# Syndrome of inappropriate antidiuretic hormone secretion as an adverse reaction of ciprofloxacin: a case report and literature review

**DOI:** 10.11613/BM.2024.010803

**Published:** 2023-12-15

**Authors:** Luka Švitek, Barbara Grubišić, Ema Schonberger, Mihaela Zlosa, Dario Sabadi, Dubravka Lišnjić, Silvija Canecki-Varžić, Ines Bilić-Ćurčić, Sanja Mandić

**Affiliations:** 1 Clinic for Infectious Diseases, University Hospital Centre Osijek, Osijek, Croatia; 2 Department of Infectology and Dermatovenerology, Faculty of Medicine Osijek, J. J. Strossmayer University of Osijek, Osijek, Croatia; 3 Faculty of Medicine Osijek, J. J. Strossmayer University of Osijek, Osijek, Croatia.; 4 Department of Endocrinology and Metabolism Disorders, Internal Medicine Clinic, University Hospital Centre Osijek, Osijek, Croatia; 5 Faculty of Dental Medicine and Health Osijek, J. J. Strossmayer University of Osijek, Osijek, Croatia; 6 Department of Pathophysiology, Faculty of Medicine Osijek, J. J. Strossmayer University of Osijek, Osijek, Croatia; 7 Department of Pharmacology, Faculty of Medicine Osijek, J. J. Strossmayer University of Osijek, Osijek, Croatia; 8 Institute of Clinical Laboratory Diagnostics, University Hospital Centre Osijek, Osijek, Croatia; 9 Department of Chemistry, Biochemistry and Clinical Chemistry, Faculty of Medicine Osijek, J. J. Strossmayer University of Osijek, Osijek, Croatia

**Keywords:** ciprofloxacin, inappropriate ADH syndrome, hyponatremia

## Abstract

Antidiuretic hormone (ADH) is secreted by the posterior pituitary gland. Unsuppressed release of ADH leads to hyponatremia. This condition is referred to as syndrome of inappropriate antidiuretic hormone secretion (SIADH). Hereby, a case report is presented on ciprofloxacin-induced SIADH. A 67-year-old male patient was examined in the emergency room with symptoms of lethargy, headache, lack of attention, and a generally depressed mood lasting for three days. One week prior, empirical antimicrobial therapy involving ciprofloxacin for prostatitis was initiated. Laboratory analysis showed no relevant abnormalities except for hyponatremia (Na = 129 mmol/L). Chronic hyponatremia, thyroid dysfunction, and adrenal dysfunction were ruled out. Serum osmolality was 263 mOsmol/kg, urine osmolality was 206 mOsmol/kg, and urine sodium was 39 mmol/L. Given that all criteria for SIADH were met, ciprofloxacin was discontinued, and fluid restriction was advised. Four days later, the patient’s serum sodium concentrations nearly normalized (Na = 135 mmol/L), and all symptoms resolved. The Naranjo Scale yielded a score of 8, supporting the likelihood of a probable adverse reaction to ciprofloxacin. This case is presented to raise awareness among clinicians about the potential of ciprofloxacin to cause even mild hyponatremia.

## Highlights

Euvolemic patient with mild hyponatremia during ciprofloxacin treatment was evaluated

No diuretic use; hypothyroidism and hypocortisolism were excluded as causes

Our findings highlight ciprofloxacin's potential role in inducing Syndrome of inappropriate antidiuretic hormone secretion

## Introduction

Vasopressin or antidiuretic hormone (ADH) is secreted by the posterior pituitary gland after synthesis in the hypothalamus (*1*, *2*). One of the roles of ADH is the regulation of tubular reabsorption of water. Antidiuretic hormone induces the synthesis of water transport proteins, thus precipitating increased water reabsorption, lowering kidney water output (*1*).

Furthermore, if ADH is not secreted as it physiologically should be, it can cause water and electrolyte imbalance. For example, if there is an unsuppressed release of ADH (in lack of usual physiological mechanisms), water intake can be exaggerated, causing intensified blood dilution resulting in hyponatremia (*1*-*3*). Therefore, the syndrome of inappropriate antidiuretic hormone secretion (SIADH) is usually recognized by clinicians when evaluating a euvolemic patient with hyponatremia.

**Figure 1:**
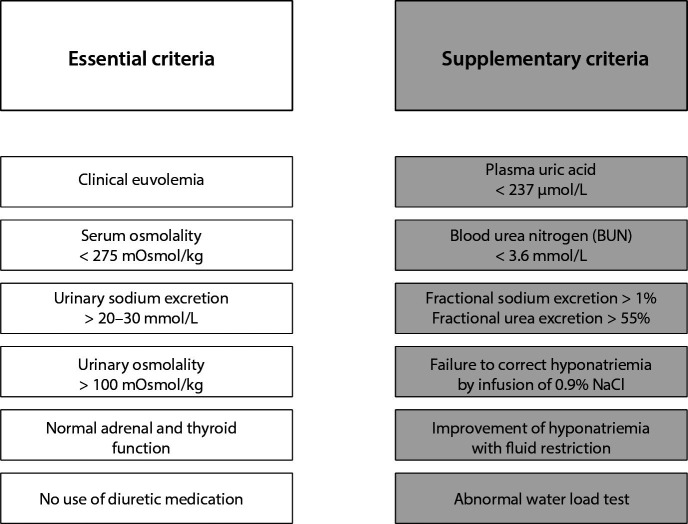
Diagnostic criteria for SIADH - adapted from Perelló-Camacho *et al.* (*3*). SIADH is a diagnosis of exclusion. All of the aforementioned essential criteria must be met, and supplementary criteria further support the diagnosis. SIADH - syndrome of inappropriate antidiuretic hormone secretion.

This syndrome was originally described in 1967 by William Schwartz and Frederic Bartter (*2*). Today we are still using their criteria for the diagnosis of the syndrome. Also, supplemental criteria are being introduced to help in establishing the diagnosis. Both criteria are presented in Figure 1 (*3*).

Ciprofloxacin is an antimicrobial agent, a member of the fluoroquinolone group, widely used for treating infections, mainly urinary tract infections (UTI), soft tissue, skin, and bone infections, gastrointestinal infections, and others. Moreover, it is a bactericidal antibiotic that inhibits bacterial DNA topoisomerase and DNA gyrase (*4*, *5*). The elimination half-life of ciprofloxacin is 4 hours (*4*).

Usually, adverse effects of this drug are nausea, vomiting, diarrhea, tendinitis, precipitation of neuropsychiatric adverse effects, and also QT prolongation (*4*-*6*). Thus, this medication should be avoided in the elderly because old age can further increase the risk of such states (*4*).

Papers reporting SIADH as a possible consequence of fluoroquinolones have been scarce, and to our knowledge, there have only been a few case reports about ciprofloxacin and other fluoroquinolones causing SIADH (*7*-*12*). Nevertheless, this adverse effect could potentially be neglected by a clinician and therefore often goes unrecognized. In other terms, it is important to report such cases to acknowledge their existence.

Syndrome of inappropriate antidiuretic hormone secretion caused or potentially caused by antimicrobial agents is rarely described in the literature. Knowledge about this adverse reaction to antibiotics is mostly derived from case reports. Apart from the fluoroquinolones mentioned earlier, SIADH can potentially be induced by other antimicrobial agents such as nitrofurantoin, trimethoprim/sulfamethoxazole, linezolid, daptomycin, azithromycin, rifabutin, and ethionamide (*13*-*20*). Hereby, a case report is presented on ciprofloxacin-induced SIADH.

## Case report

A 67-year-old male patient was examined in the Infectious diseases emergency room, where he was directed during the triage process due to suspected unresolved urinary tract infection. During the examination, the patient reported symptoms of lethargy, headache, difficulty with concentration, lack of attention, and a generally depressed mood lasting for three days. One week prior, the patient was examined by an urologist due to urinary retention, which resulted in the placement of a permanent urinary catheter. Additionally, the patient had been started on empirical antimicrobial therapy with ciprofloxacin due to an enlarged and painful prostate.

From the personal medical history, it is noted that the patient has prostate hyperplasia, chronic gastritis, and depressive syndrome. He has been on regular therapy for years, which includes pantoprazole, sertraline, alprazolam, and tamsulosin. The patient did not report any malignancy that he was aware of. Physical examination revealed a gentle upper limb tremor and no other abnormalities; the patient was euvolemic.

Laboratory analysis, chest X-ray, and urine analysis were performed and there were no clinically relevant abnormalities that could cause such symptoms besides moderate hyponatremia (Na = 129 mmol/L). Blood urea nitrogen was 2.4 mmol/L. Laboratory findings are presented in Table 1.

**Table 1:** Patient’s laboratory findings supporting the diagnosis of SIADH

**Analyte (unit)**	1^st^ analysis results*	**2^nd^ analysis results^†^**	**RI**
Sodium (mmol/L)	129 (L)	135 (L)	137-146
Potassium (mmol/L)	3.9	4.2	3.9-5.1
Chloride (mmol/L)	96 (L)	NP	97-108
Urea (mmol/L)	2.4 (L)	2.8	2.8-8.3
Creatinine (µmol/L)	66	77	64-102
Osmolality (mOsmol/kg)	263 (L)	NP	275-290
Urine osmolality (mOsmol/kg)	206	NP	50-1200
Urine sodium (mmol/L)	39	NP	20-400
Thyroid-stimulating hormone (mIU/L)	1.047	NP	0.350-4.940
Thyroxine (pmol/L)	12.30	NP	9.01-29.05
Cortisol - morning (nmol/L)	400	NP	101-536
Adrenocorticotropic hormone - morning (pmol/L)	4.2	NP	1.6-13.9
*Samples for analysis were taken during the initial patient examination. ^†^Samples for analysis were taken 4 days after the initial examination and discontinuation of ciprofloxacin. Although the results of urine analyses fall within the reference values, they meet the criteria for SIADH. Sodium, potassium, chloride, urea and creatinine were determined on the AU680 (Beckman Coulter, Brea, USA). Osmolality was measured using Osmo Station OM-6060 analyzer (Arkray factory, Shiga, Japan). Thyroid-stimulating hormone, thyroxine and cortisol were determined on the Alinity i analyzer (Abbott Diagnostics, Abbott Park, USA). Serum sampling was performed using 4 mL BD Vacutainer CAT, Clot Activator Tube (Becton, Dickinson and Company, Plymouth, United Kingdom). Urine sampling was performed using URINtainer (F.L. Medical, Torreglia, Italy). Adrenocorticotropic hormone was sampled using 3 mL BD Vacutainer K2EDTA (Becton, Dickinson and Company, Plymouth, United Kingdom) and measured on the Cobas 6000 (Roche Diagnostics GmbH, Mannheim, Germany). RI - reference intervals. NP - not performed. L - below the lower limit of the reference interval. SIADH - syndrome of inappropriate antidiuretic hormone secretion.			

Less than a month prior to these events, the patient underwent a routine laboratory blood analysis that showed normal concentrations of serum sodium (Na = 138 mmol/L). The aforementioned ruled out chronic hyponatremia so further analysis and evaluation were to be done.

Serum and urine osmolality, as well as urine sodium concentrations, were measured. Serum osmolality was 263 mOsmol/kg, urine osmolality 206 mOsmol/kg, and sodium in urine was 39 mmol/L. Next, thyroid and adrenal function were checked, and all results (thyroid stimulating hormone, thyroxine, cortisol, and adrenocorticotropic hormone) fell within the reference range (Table 1). Consequently, ciprofloxacin-induced SIADH was suspected. Hospital admission for the patient was offered but respectfully declined.

Since all the criteria for SIADH were met, the chosen treatment regimen involved discontinuing the causative medication and advising fluid restriction, following the recommended management guidelines for SIADH (*21*, *22*). Considering that there were no manifestations of severe hyponatremia in laboratory findings or clinically, and the acute decrease in serum sodium concentration did not exceed 10 mmol/L, hypertonic saline infusion was not selected for treatment at this time. The patient remained under physician control and continued with regular policlinic check-ups.

The patient’s ciprofloxacin treatment was discontinued, and trimethoprim-sulfamethoxazole was administered instead. This decision was made following the Croatian national guidelines, which recommend a four-week course of treatment for prostatitis (*23*).

Four days later, serum sodium concentrations in the patient almost reached normal values (Na = 135 mmol/L), and all of the patient’s symptoms resolved. No other medical interventions were made, besides discontinuing ciprofloxacin and advising water intake restriction.

The Naranjo adverse drug reaction probability scale was performed, and a final score of 8 supports the likelihood of a probable adverse ciprofloxacin reaction (*24*).

Written informed consent was acquired from the patient for the purpose of publishing this case report.

## Discussion

Considering all the circumstances in this case, SIADH was probably an adverse reaction to ciprofloxacin. The patient presented with mild to moderate hyponatremia symptoms and did not have a history of chronic hyponatremia. The only contrasting factor, not previously present, was ciprofloxacin intake. Also, all of the symptoms were revoked, and sodium serum concentrations returned to normal values after ciprofloxacin discontinuation.

As stated, other common causes of potential euvolemic hypoosmolality, hypothyroidism and hypocortisolism, were excluded.

It is also essential not to neglect that the patient was taking sertraline, an antidepressant that belongs to selective serotonin reuptake inhibitors (SSRIs) which are also known to cause SIADH (*25*, *26*). However, in this case, it is unlikely for sertraline to cause acute hyponatremia since the patient has been taking it for multiple years without recent dosage changes. On the other hand, sertraline is metabolized by human cytochrome P450 (CYP) isoforms, the same as ciprofloxacin. However, ciprofloxacin is mainly metabolized by CYP1A2 and is an inhibitor of that enzyme, while sertraline is metabolized by many CYP isoforms (*4*, *27*). Sertraline concentration is mainly affected by CYP2C8/9 and CYP2C19 inhibition. Studies have shown only minimal effect (if any) of CYP1A2 inhibition on sertraline serum concentrations (*27*). Therefore, ciprofloxacin use should not precipitate the rising of sertraline serum concentrations. All of the stated still advocates reported SIADH being caused by ciprofloxacin intake.

The effect of ciprofloxacin on ADH secretion could be mediated by involving GABA (γ-aminobutyric acid) receptors. It has been reported that the activation of GABAergic inputs inhibits ADH-secreting neurons (*28*, *29*). Additionally, it has recently been described that fluoroquinolones inhibit GABA receptors in the central nervous system through direct pharmacological activity (*30*). Since ciprofloxacin is an antibiotic that crosses the blood-brain barrier, SIADH could be caused by the proposed mechanism (*4*).

Considering all the previously reported cases of SIADH caused by ciprofloxacin, including this one, the duration of exposure to ciprofloxacin before developing SIADH symptoms varies between two to five days (*7*-*9*). This adverse reaction is typically observed in elderly individuals over 65 years of age (ranging from 67 to 84 years of age), as it has also been reported with other antimicrobials (*31*). Caution is necessary when prescribing medication to the elderly due to age-related physiological changes affecting the pharmacologic profile. Therefore, elderly patients should receive comprehensive information regarding the risk of adverse effects development, which remains low, but higher than in younger individuals. All of the reported cases had met every one of the essential criteria and some of the supplemental criteria for SIADH (*3*, *7*-*9*).

Comparing the severity of clinical manifestation with other reported cases of ciprofloxacin-caused SIADH, the observed patient had mild to moderate hyponatremia, while other described patients had severe hyponatremia (serum concentrations of sodium 114 mmol/L or less), presenting with bradyphrenia, seizures, and/or coma (*7*-*9*). The motive for usually reporting only more severe cases could be that mild to moderate hyponatremia is easily overlooked as a reason for further investigation due to its tendency to resolve in the clinical course of the disease. Hence, this case is presented in order to draw the attention of clinicians in recognizing ciprofloxacin as a possible causative agent of even mild hyponatremia.

In conclusion, with this case report we want to emphasize the importance of adverse drug reactions in everyday clinical work, as they are often neglected. Fluoroquinolones, as a group of antimicrobials that are potent against many infective agents, are widely used. Therefore, special attention should be given to considering the adverse effects of this medication group.

## Data Availability

All data generated and analyzed in the presented study are included in this published article.
